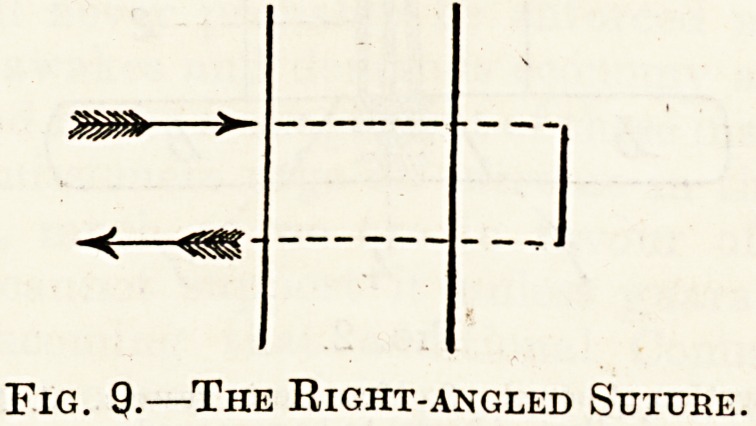# Graduate Teaching Facilities in London Hospitals

**Published:** 1906-09-15

**Authors:** 


### Graduate Teaching Facilities in London Hospitals.

### WEST LONDON POST-GRADUATE COLLEGE.

The following notes are from a lecture on practical surgery in injuries of the abdomen which was a continuation of one given a few days previously by Mr. Aslett Baldwin, F.R.C.S., Teacher of Operative Surgery Post Graduate College, to a class of graduates attending the Graduate College in connection with the West London Hospital at Hammersmith. It is illustrative of the practical methods of instruction usually adopted at the West London College, and its record here cannot fail to be of great service to all practitioners. The illustrations are prepared from sketches actually made on the blackboard.

### Injuries of the Omentum.

A hæmatoma should be removed because it is so liable to get adherent to the bowel-wall and cause intestinal obstruction; it should be removed by means of "interlocked" ligatures. After hæmatoma is removed the sides of the gap are brought together with sutures. If there is a transverse cut the vessels are ligatured and you discover if any of the omentum is likely to die; if so, it must be removed. In ligaturing the omentum, be careful not to go too near the large intestine, but leave a good stump, at least an inch long, on account of the danger of intestinal obstruction taking place later on.

Fig. 1.

1. Hæmatoma in omentum. 2. Portion of omentum needing removal

### Injuries of the Intestine and Mesentery.

In these cases it is extremely important to make up the mind when an injury is found in the mesentery whether the bowel is going to live. If the mesentery is torn across the very maximum of denuded bowel that is safe to leave is two inches. If there is over two inches the bowel must be excised. In doing this the bowel is cut rather obliquely so as to ensure a larger opening and a smaller degree of contraction. A circular opening tends to cause a subsequent stricture of the bowel. The end-to-end method is better than the lateral anastomosis except where you have to unite a small to a large viscus, as in the case of the ileum and cæcum. The most difficult part to unite is the mesenteric border of the bowel, because the bowel is not surrounded by peritoneum, at this part leakage is most likely to take place, and the muscular coat tends to retract (fig. 4). Before the bowel is divided it must be clamped a few inches away from the proposed section on each side. If clamps are not available, the bowel can be surrounded with fine drain tube. It is passed through a small hole bored through the mesentery close to the bowel, then tied once and held by Spencer Wells forceps.

Fig. 2.

1. Oblique section of bowel. 2. Mesenteric vessels. 3. Mesentery. 4. Part of bowel to be removed.

Fig. 3.

1, 2, 3. As in fig. 2. 4. Segment of bowel excised.

Fig. 4.

1. Peritoneal coat of bowel. 2. Bowel. 3. Mesentery. The arrow points to the mesenteric border of the bowel, where it is not covered with peritoneum.

Fig. 5.—Method of using Drain Tube and Spencer-Wells Forceps Instead of Bowel Clamps in Resection of Intestine.

1. Drain tube. 2. Spencer Wells forceps. 3. Hole in mesentery. *M.* Mesentery.

The muscular coats are sutured first at the mesenteric border for perhaps half an inch, making sure that the two muscular coats are got together. After that begin again with a continuous suture through all the coats until half-way round, doing that suturing in a continuous circle, working from inside the bowel. When half round take the stitch through twice to avoid dragging the bowel up. In stitching the second half work from the outside. In this way a continuous suture goes round through the coats of the

Fig. 6.—Muscular Coat of Bowel Joined at the Mesenteric Border.

1. Mucous coat. 2. Peritoneal coat. 3. Muscular coat. 4. Suture joining muscular coat at mesenteric border. *M.* Mesentery. *B.* Bowel.

bowel and a special suture at the mesenteric border. This first suture serves also to avoid the risk of not getting the mesenteric borders opposite each other.

Fig. 7.—To Show Method of Continuous Suturing.

Then go round the bowel with another continuous suture, taking all the coats except the mucous. Should the stitches look like not holding it is well to reinforce them by (1) either stitching a piece of omentum round, leaving it attached; or (2) you can cut the piece of the omentum completely off and stitch it round the bowel; this gives least chance of strangulation. New vessels come in from the peritoneum, or in the large bowel the appendices epiploical may be used. The redundent mesentery should be folded over and fixed by sutures; it need not be removed. If the anastomosis is some distance away from the stomach the patient may be fed as soon as the vomiting is over. The food must not produce gas. Salol gr. x. is given twice daily to prevent its formation in the bowel. It is decomposed in the bowel into salicylic acid and phenol. Instead of milk the patient should get grape juice or albumen water (white of eggs, sugar, and water, flavoured with lemon juice). If fresh grapes are not obtainable raisins may be stewed for one hour in twice their bulk of water and the fluid given diluted, adding a little lemon-juice if it is too sweet. These are better than milk, which tends to ferment and distend the bowel with wind. If the bowel injury is near the stomach, or if the vomiting is pressing, the patients are fed by the rectum for two or more days. In the latter case the patient is given 4 oz. of hot water to drink, containing 30 gr. of bicarbonate of soda and 8 minims of essence of peppermint, or the stomach is washed out through a tube.

In regard to drainage, it is most valuable when there is doubt as to the safety of the stitches, should they drag or should the intestine be atrophied and likely to give way. Gauze drainage is the best; it makes a tract along which the bowel contents find their way. Should a fæcal fistula form by the bowel giving way it will heal up. The difficulty is that the contents cause a good deal of skin irritation. Zinc ointment on lint, applied zinc side downwards against the whole of the belly wall, will relieve that condition and the fistula will close with few exceptions in two or three weeks.

### Injuries of the Kidney.

The kidney is very commonly damaged in injuries to the loin. One has to estimate whether the injury is merely contusion of the kidney; whether it is ruptured and the urine extravasating into the loin, or whether along with that the peritoneum has been ruptured and the extravasated urine entering the abdominal cavity. In all these cases there is considerable shock.

(*a*) If the kidney is contused blood will appear in the urine, no swelling in the loin, no pain in the belly, no distension of the bowel; no meteorism, no peritonitis, no fluid collection in the abdomen. The treatment consists of hypodermic styptics, ergot or adrenalin, rest in bed, perhaps an ice-bag to the loin, when the shock has passed off.

(*b*) If swelling appear in the loin with a rising temperature and pain, it means that the kidney is ruptured and urine extravasating. In that case put the patient on the sound side cut down, see what the condition is, and clear out the blood and urine, because if it is left a large abscess will form. In late years the surgery of the kidney is more conservative than it was, and any useful part that can be saved should be left. The kidney can be split in half and sutured and do perfectly well. It is sutured by means of right-angled sutures.

Fig. 8.

1. V-shaped section to remove. 2. Damaged portion of kidney.

Part of the kidney may be pulped. This can be cut out by means of a V-shaped incision, the two ends being afterwards stitched together with a right-

Fig. 9.—The Right-angled Suture.

angled suture (fig. 9). If the main vessels are bleeding, the kidney must be removed. Never attempt to put a ligature round the whole pedicle of the kidney, it is so apt to slip off. Ligature the vessels and the ureter separately, wash out with a weak mercuric lotion, and, in any case, whatever is done, it is well to put in a drain.

(c) If abdominal swelling and tenderness, vomiting, dullness of the flank, extending later on to both flanks, should occur, it points to rupture of the peritoneum and extravasation into the abdomen. That calls for operation at once. The best thing to do in that case is to go through the belly cavity outside the semilunar line. Sponge out the blood and urine, get down to the kidney and investigate. Keep to the outer side of the colon. If you take the inner side the blood-supply of the colon is cut off and necrosis would follow. Save the kidney if possible. Completely suture up the rent in the peritoneum and close the abdominal cavity. As it is necessary to drain, make an incision through the loin down to the kidney.

### Rupture of the Bladder.

In injury to the pelvis the urethra may be ruptured at the same time as the bladder. It is important to make out whether the rupture is extra- or intra-peritoneal. In these cases the pelvis may be fractured. Before beginning investigations it is advisable to irrigate the urethra with a back-flow catheter. Having cleaned the meatus the urethra is washed for some distance. A metal catheter is used, lubricated with glycerine and mercury 1 in 1,000. If the bladder is ruptured you may find the point of the catheter coming through the bladder into the abdominal cavity, or the point may just be felt through the bladder in the cellular tissue, where only slight movement of the catheter is possible, this means extra peritoneal rupture. To settle the matter, after drawing off the fluid, inject six ounces of warm boric solution into the bladder, and if it all comes back it is not an intra-peritoneal rupture, but there may be an extra-peritoneal one. In an extra-peritoneal rupture there will be in time a tender swelling rising above the pubis—due to extravasation of urine. If urine extravasates into the peritoneal cavity, fluid in the flanks and tenderness are present, and later on peritonitis. A supra-pubic operation has to be done, and an exploration made behind the pubis for the rupture. Suture is up if it is within reach, and drain supra-pubically. If the rupture is into the peritoneum, that must be opened by enlarging the incision. The Trendlenberg position may be employed after mopping up the blood and urine and Lembert's sutures used for the viscus. In the case where the rupture cannot be got at, drain supra-pubically. If there has been rupture with extravasation into any part extra-peritoneally, drain the tissues well.

The question of leaving the catheter in or passing it at intervals of four to six hours. If the urethra has been thoroughly cleansed by the back-flow catheter, it is better to leave the catheter in attached to a long tube conducted under the bed into an antiseptic solution, and give the patient urotropin, gr. viij., three times a day after food. If a larger dose than that is given, the digestion is apt to be upset. There is less risk of sepsis in keeping the catheter in than in passing it continually. Its nose should only just lie within the bladder. It can be removed in six days.

### Rupture of the Urethra.

In either rupture of bladder or of urethra urgency of micturition is usual, and the patient should be warned against straining, to prevent extravasation. If there is rupture of the urethra, blood will pass out through the meatus. It should be very carefully cleansed and washed out. The catheter may or may not pass. Sometimes it can be passed quite easily under an anæsthetic and left in for several days. If it is impossible to get the catheter in, put the patient in the lithotomy position, pass the staff, cut down and expose the torn ends of the urethra. To find the proximal end is a difficulty which increases very much with time. If there is urine in the bladder it will assist in this. Perhaps by pressing above the pubis some of the urine may be pressed out and you can see the outlet. If this is not sufficient, the bladder must be opened above the pubis and a large probe passed from within outwards. Having found the opening, a No. 12 gum-elastic catheter should be passed through the penis and into the bladder. This is much easier said than done. A string may be attached to the bladder probe and attached to the gum-elastic, which in this way may be assisted in entering the proximal aperture. Having got the catheter through and into the bladder, the urethra is sutured round the catheter—often extremely difficult to do on account of irregularity of the torn edges. The sutures should be made with fine catgut. The skin wound in the perineum should be left entirely open. Suture the other tissues, but leave the skin-wound. It comes together when the lithotomy position is finished, and if leakage takes place the urine escapes and extravasation does not take place.

## Figures and Tables

**Fig. 1. f1:**
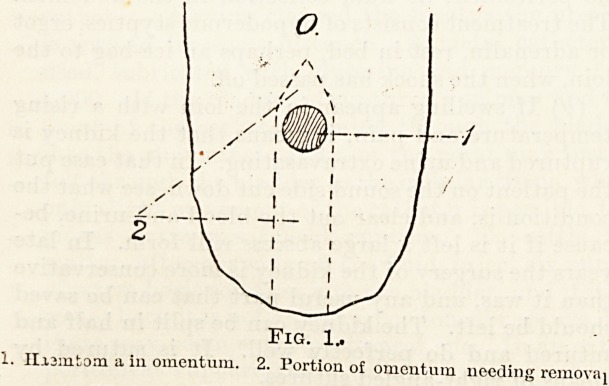


**Fig. 2. f2:**
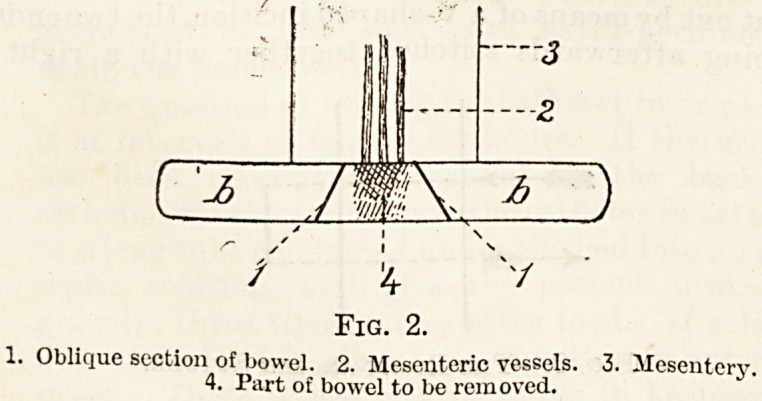


**Fig. 3. f3:**
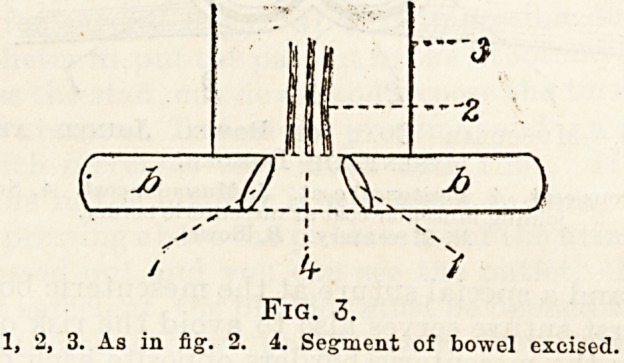


**Fig. 4. f4:**
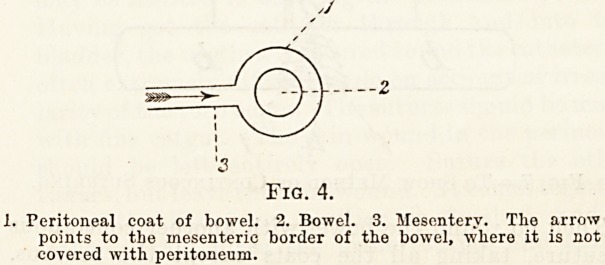


**Fig. 5. f5:**
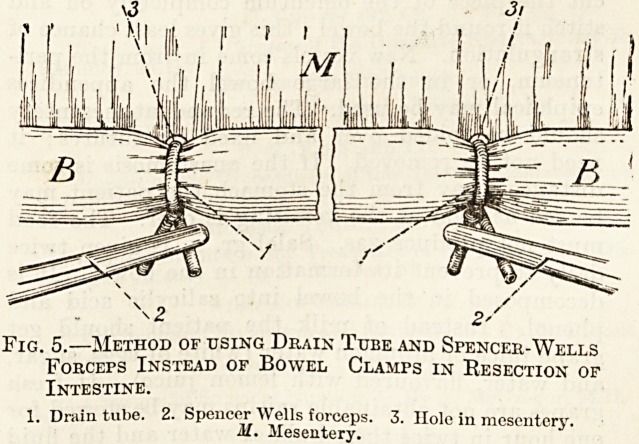


**Fig. 6. f6:**
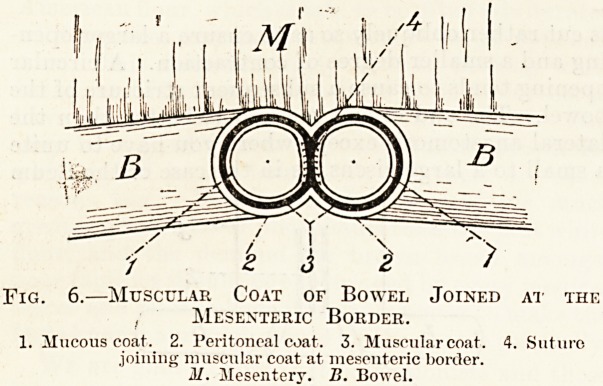


**Fig. 7. f7:**
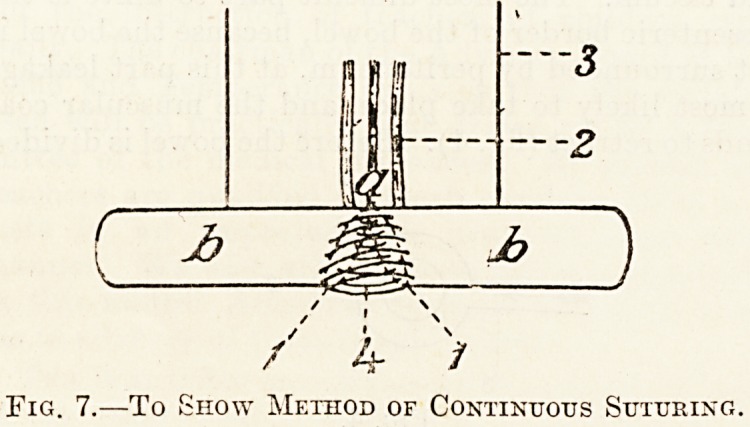


**Fig. 8. f8:**
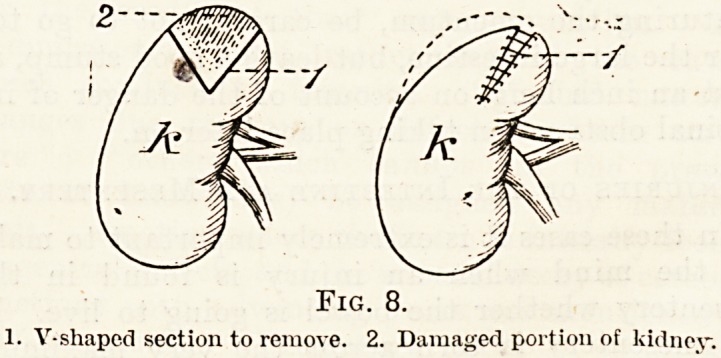


**Fig. 9. f9:**